# Alginate–Gelatin Hydrogel Scaffolds; An Optimization of Post-Printing Treatment for Enhanced Degradation and Swelling Behavior

**DOI:** 10.3390/gels9110857

**Published:** 2023-10-28

**Authors:** Christina Kaliampakou, Nefeli Lagopati, Evangelia A. Pavlatou, Costas A. Charitidis

**Affiliations:** 1 RNanoLab, Research Unit of Advanced, Composite, Nano Materials & Nanotechnology, School of Chemical Engineering, Zografos Campus, National Technical University of Athens, 9 Heroon, Polytechniou St., 15780 Athens, Greece; kaliabakou@chemeng.ntua.gr; 2 Laboratory of Biology, Department of Basic Medical Sciences, Medical School, National and Kapodistrian University of Athens, 11527 Athens, Greece; 3 Biomedical Research Foundation, Academy of Athens, 11527 Athens, Greece; 4 Laboratory of General Chemistry, School of Chemical Engineering, National Technical University of Athens, Zografou Campus, 15772 Athens, Greece; pavlatou@chemeng.ntua.gr

**Keywords:** optimization DoE, post-printing treatment, scaffolds, degradation, swelling

## Abstract

The generation of 3D structures comprises three interlinked phases: material development, the printing process, and post-printing treatment. Numerous factors control all three phases, making the optimization of the entire process a challenging task. Until now, the state of the art has mainly focused on optimizing material processability and calibration of the printing process. However, after the successful Direct Ink Writing (DIW) of a hydrogel scaffold, the post-printing stage holds equal importance, as this allows for the treatment of the structure to ensure the preservation of its structural integrity for a duration that is sufficient to enable successful cell attachment and proliferation before undergoing degradation. Despite this stage’s pivotal role, there is a lack of extensive literature covering its optimization. By studying the crosslinking factors and leveling the post-treatment settings of alginate–gelatin hydrogel, this study proposes a method to enhance scaffolds’ degradation without compromising the targeted swelling behavior. It introduces an experimental design implementing the Response Surface Methodology (RSM) Design of Experiments (DoE), which elucidated the key parameters influencing scaffold degradation and swelling, and established an alginate ratio of 8% and being immersed for 15 min in 0.248 M CaCl_2_ as the optimal level configuration that generates a solution of 0.964 desirability, reaching a degradation time of 19.654 days and the swelling ratio of 50.00%.

## 1. Introduction

Direct Ink Writing (DIW) is an Additive Manufacturing (AM) method that enables the creation of complex 3D structures with intricate designs and various material compositions for biomedical applications [1]. The rapid progress in the development of innovative biomaterials that are processable for extrusion-based AM methods like DIW allows for strong biological interactions, eventually facilitating the cultivation of 3D artificial tissues [2]. In DIW, a viscoelastic ink is extruded through a deposition nozzle, layer by layer, to construct scaffolds [3]. Unlike other manufacturing methods, DIW-based scaffold development allows for the precise and controlled placement of biomaterials, highlighting its potential as a means to produce innovative and adaptable grafts which are suitable for various types of tissues [4]. The cost-effectiveness, ease of use, and capability to blend multiple materials in a single manufacturing step have gathered significant interest from various research teams worldwide, leading to extensive advancements in this cutting-edge technology. The precision of DIW can address the need of Tissue Engineering (TE) for artificial tissues with embedded vascular networks and establish directed vascularization, increasing the compatibility and longevity of the printed structures [5]. Up until now, much effort has been made in achieving a high precision in geometry fidelity by optimizing materials’ ink properties and the printing process. However, one of the most crucial factors in developing functional 3D systems is their ability to preserve their structural integrity for a sufficient duration, as guided by the specific requirements of the target tissue. In the case of guided vascularization, studies suggest that a three-week (21-day) cell culture is the appropriate timeframe for vessel formation [6].

This stage, which is referred to as the post-printing treatment, is mainly governed by the crosslinking process which, in turn, is determined by the ink’s composition. In the present study, based on the printing approach and the need for in situ gelation, to ensure that the printed products maintain their structural integrity and stability after printing, alginate–gelatin ink was selected. This hydrogel ink possesses the desired properties for efficient use in DIW, as presented in previous results, and shows great compatibility with biological systems [7]. Alginate-based biomaterial inks have gained popularity, as alginate is a biodegradable and biocompatible polysaccharide derived from brown algae, and is capable of forming a gel through ionic crosslinking [8]. It has been employed for printing vascular tissue, bone, and cartilage-like structures. Gelatin is another well-researched biomaterial used as ink for DIW. Gelatin provides arginine–glycine–aspartic acid (RGD) cell adhesion motifs in alginate–gelatin composite hydrogel, imparting bioactivity and enhancing cell adhesion capabilities. In addition, these oligopeptide sequences containing RGD peptide also result in more favorable scaffold degradation compared to pure alginate [9,10]. Thus, although alginate lacks the desired bioactive properties and gelatin has limitations in terms of mechanical strength, the right combinations of these materials can lead to biomaterial inks that are suitable for scaffolds that promote directed vascularization [11].

Post-printing treatment is necessary for both alginate and gelatin to acquire good mechanical properties and stability. First, by cooling down the printed structures (20 °C), the physical crosslinking of the temperature-dependent gelatin is activated. Then, these pre-crosslinked samples are soaked in Ca^+^ solution. The interaction between multivalent cations and the glucuronic blocks in alginate leads to ionic crosslinking of the alginate particles, resulting in the formation of a 3D network and enhancing the mechanical properties of the created scaffold [12]. The crosslinking of hydrogels has the effect of preserving their shape even though they can absorb significant amounts of fluids and swell. Thus, a necessary equilibrium must be established between the degradation rate and the ratio of swelling when introducing a scaffold in in vitro and in vivo applications [13].

However, when studying the post-printing treatment, and aiming to decrease the degradation rate, the optimization process should take into consideration that the scaffold’s functionality might be decreased due to four different reasons related to the handling of the scaffold following the printing. Firstly, when sodium alginate-based hydrogels, crosslinked with calcium ions, are exposed to physiological conditions, a process occurs where divalent calcium ions are gradually replaced by monovalent sodium ions present in the degradation medium [13,14]. Secondly, when alginate–gelatin scaffolds are incubated in culture media, temperature-dependent gelatin is gradually dissolved and released from the scaffold due to high temperature (37 °C), leading to hypoxia affecting cell viability. However, the inclusion of crosslinked sodium alginate matrices, as well as the interaction between alginate and gelatin, enhances the thermal stability of gelatin and slows down the release of gelatin from the printed scaffolds when maintained at 37 °C [15]. Furthermore, densely arranged hydrogels could pose a substantial obstacle to the diffusion of various substances (such as protein molecules, gases, growth factors, and metabolic waste) between the enclosed cells and the surrounding culture medium. This confinement of cells could lead to diminished cell viability. For this reason, the swelling capacity of the scaffolds is highly important [16]. Last, but not least, after implantation, a distinct reduction in size can lead to detachment, and an uncontrolled increase in the substrate could result in severe inflammation or patient discomfort. The shaping process can be accurately managed through specific parameters during fabrication. Therefore, it is of high importance to address the deformation that occurs after the treatment [17].

Thus, through the examination of the factors related to crosslinking, an approach that improves the degradation of scaffolds while maintaining their functionality needs to be developed. In the existing literature, when aiming to decrease the scaffold’s degradation rate in order to reach the desired time interval without losing its structural integrity, emphasis is mainly given to studying the parameters of the crosslinking stage (alginate ratio, crosslinker concentration, immersion time) in correlation with other scaffold properties, such as its stiffness and mechanical properties [18]. For instance, Naghieh at al. showed that both the duration of the immersion and the concentration of the crosslinker employed are critical factors that significantly influence the mechanical properties in 3D bioplotted alginate scaffolds [19]. Bahrami et al. demonstrated that the 4% alginate scaffold experienced substantial weight loss in various solutions, and its dissolution rate was significantly greater than that of the 8% and 16% alginate scaffolds in all of the tested solutions, while 16% alginate scaffold, due to its density, presented a very low degradation rate [20]. Furthermore, Sonaye et al. showed that increasing the concentration of crosslinking and the amount of alginate in the printed scaffolds improved the swelling capacity and reduced the degradation rate [21]. Specifically, they identified that an optimal crosslinking concentration of 500 mM CaCl_2_ and an alginate content of 12% (*w*/*v*) resulted in scaffolds with high swelling (70%) and low degradation rates (28%). This research team established a connection between crosslinking parameters and scaffold characteristics, such as stiffness, swelling capacity, and degradation rate, to create scaffolds suitable for durable skeletal muscle tissue constructs in tissue engineering applications. Finally, Lei et al. suggested treating alginate/gelatin scaffolds with CaCl_2_ and CaCl_2_–EDC solutions when targeting minimal swelling (50%) throughout the post-treatment period. Their findings confirm that the deformation occurring after treatment can be controlled through a crosslinking process [17].

In addition, until now, the state of the art, including our team’s previous work [22], in Direct Ink Writing (DIW) has primarily relied on the Design of Experiments (DoE) to optimize the initial two stages, which encompass the material processability and printing process parameters. There is also a limited number of studies optimizing some scaffold properties, like porosity, based on material composition [23]. Notably, the existing literature lacks any evidence of a similar methodology applied to the subsequent post-printing stage. However, it is imperative to extend the application of the DoE to optimize the post-printing treatment phase, given that it is influenced by numerous factors which are often overlooked (UV treatment, culture media), resulting in inefficient resource utilization through trial-and-error approaches [24]. Furthermore, the implementation of an experimental design becomes crucial to identify the most functional compromise within the 3D system, focusing on key responses such as the degradation behavior and swelling ratio. Such a design would not only illuminate the interactions among the post-printing treatment factors but also expedite the development of a predictive model for enhanced efficiency.

The primary objective of this research is to bridge this gap and offer insights into optimizing the post-printing treatment of alginate–gelatin hydrogel, building upon our team’s prior published research [22], which focused on optimizing ink processability and the printing process. Specifically, this study seeks to improve the degradation characteristics of scaffolds while maintaining their swelling capacity. To address these objectives, an experimental design is introduced that employs Statistical Response Surface Methodology (RSM DoE). This approach aims to systematically uncover the key parameters that govern post-printing treatment and establish their optimal levels. In this context, we developed alginate–gelatin scaffolds through DIW to explore and optimize the effect of crosslinking on their biodegrability and deformation. The ink formulation and printing parameters were adjusted based on the results of the previous work through the corresponding three-step optimization method [22]. The combination of the levels that we judged to be optimal for each parameter has resulted in the printing of rectangular scaffolds with a high geometry fidelity. Following, via RSM DoE, we studied which post-printing factors play the most crucial role in scaffolds’ functionality and, after defining the optimal levels, we investigated the properties of the scaffolds that were treated with the conditions identified as optimal by the experimental design. Thus, the current paper presents an innovative approach that addresses the critical yet underexplored stage of scaffold post-printing treatment. By implementing a novel approach that utilizes RSM design, this study concurrently optimizes two vital scaffold properties: degradation and swelling behavior. These responses are inherently challenging to co-currently optimize, as maximizing one often leads to a decrease in the other and vice versa. Reaching the targeted values for both responses would improve the total functionality of alginate–gelatin scaffolds.

## 2. Results and Discussion

### 2.1. Scaffolds Development

After the development of the three distinct alginate–gelatin ink configurations (4/4 (%), 6/4(%) and 8/4 (%)), the successful Direct Ink Writing of the scaffolds for use in the suggested screening tests was subsequently undertaken. The four samples were printed in triplicate and each of them was then methodically treated in accordance with the specified conditions, as determined by the selected leveling of the post-printing factors, in order to record the variance in the degradation time and swelling ratio results. For the assessment of the scaffolds’ shape fidelity and structural integrity, precise measurements were taken, including their dimensions and the diameter of their individual strands, via ImageJ (National Institutes of Health, Bethesda, MD, USA) software. The term ‘material strand’ refers to the diameter of the deposited material. In this context, it is compared to the theoretical value, which is defined by the tip’s diameter (0.41 mm in this case). This comparison serves to define the printability of the ink based on the following equation (Equation (1)):

(1) $$\mathrm{Strand}\;\mathrm{Printability}:\;1-\frac{Ds-Dexp}{Ds}$$

where *D_s_* is the theoretical strand diameter and *D_exp_* is the experimental strand diameter [22].

Furthermore, their dry weight was determined and recorded for further use in the deformation analysis. The results for Samples 1–4 are presented in Table 1, providing a comprehensive overview of the screening scaffolds’ characteristics. An ideal printability value of 1 signifies a perfect match between the experimental strand and the theoretical model, demonstrating the precise deposition of material. The results presented in Table 1 reveal a printability trend for samples with a fixed gelatin ratio of 4% and varying alginate content within the 4–8% range. Specifically, it becomes evident that, as the alginate ratio increases, as shown by Sample 4 with 8% alginate, printability significantly improves. This observation is consistent with prior findings in the field [22,25]. As presented, higher printability values correlate with a reduced scaffold mass and smaller dimensions, indicating a more efficient printing process. This is attributed to the deposition of less material in each layer, resulting in superior shape fidelity.

### 2.2. Screening Tests Characterizations Results

#### 2.2.1. Degradation Behavior

**Figure 1:**
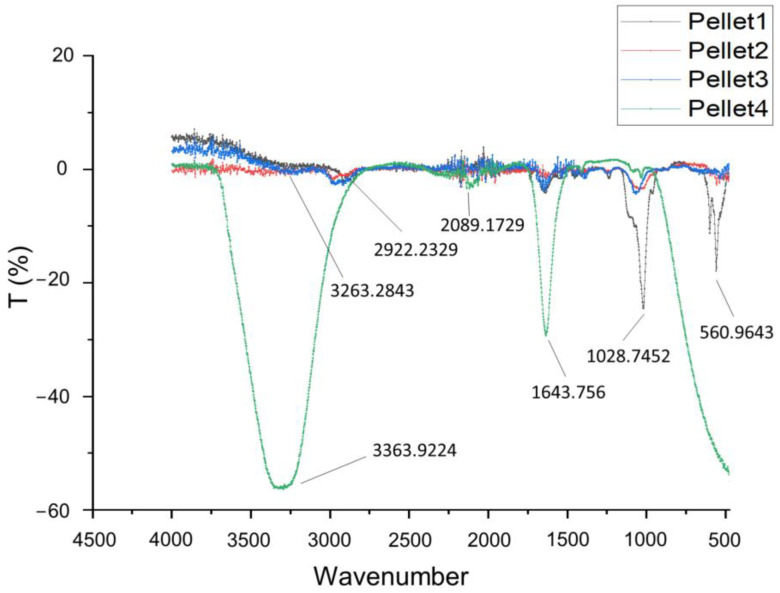
FT-IR spectra (4500–450 cm^−1^) of centrifuged culture media for the four screening samples.

Figure 1 displays the FT-IR (Fourier-transform infrared spectroscopy) spectra of the pellets that were collected after the centrifugation of the media in which the screening scaffolds (Samples 1–4) were immersed for 7 days. As confirmed in Figure 1, the amino group in gelatin and the carboxyl acid group in alginate exhibited electrostatic attraction, leading to the formation of the alginate–gelatin network backbone [26]. The small absorption peaks at 3263 cm^−1^ are characteristic of sodium alginate for –OH stretching [27]. However, with regard to this absorption band, concerning the stretching vibration of the N–H group bonded to O–H group, the increase in intensity is reported to be characteristic of an increase in intramolecular bonding attributed to CaCl_2_ crosslinking [17,28]. Another small peak at 2922 cm^−1^ which is partially overlapped with the peak for –OH stretching is assigned to N–H stretching from gelatin or C-H stretching from alginate [29]. The distinctive peak at 1643 cm^−1^ is associated with the stretching vibrations of the asymmetric and symmetric -COO- groups, as found in sodium alginate infrared spectra (1635 cm^−1^) peaks at 1640 cm^−1^ for C=O, from gelatin [27]. Another characteristic peak of alginate at 560 cm^−1^ clearly indicates its dissolution in the culture media. The absorption peaks at 1028 cm^−1^ are strongly indicative of dissolute gelatin (Amide III) [26] and have coupled with stretching vibrations of C-O from pure sodium alginate, which is recorded at 1043 cm^−1^ [29]. Moreover, the strong peaks exhibited at 3363 and 1643 cm^−1^, as well as the wide and small peak at 2089 cm^−1^, are characteristic peaks of the water spectrum. According to the obtained spectra, it can be concluded that Sample 1, with strong characteristic peaks of alginate and gelatin, was totally dissolved in the culture medium by Day 7, demonstrating a high degradation rate. Samples 2 and 3 exhibited peaks that were smaller but also indicative of alginate and gelatin, leading to the conclusion that those samples demonstrated a medium degradation rate. Finally, the spectrum of Sample 4 is characterized by water-indicative peaks, which means that no dissolute alginate or gelatin was detected in the culture medium. Thus, Sample 4 exhibits a small degradation rate, verifying that the selected levels for the optimization DoE indicate a sufficient range of degradation rates.

**Figure 2:**
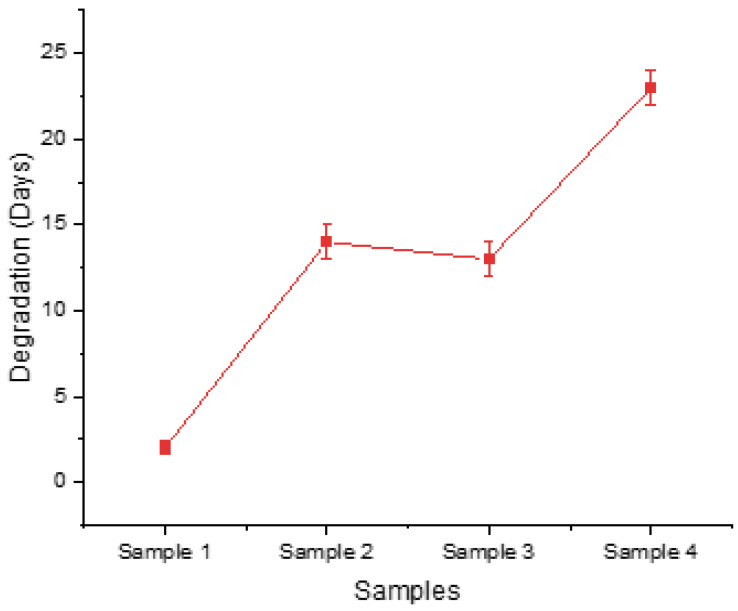
Time needed for each screening sample to maintain its structural integrity before dissolving in culture medium.

These findings are also confirmed in Figure 2, where there is evidence that the configuration of low levels led to rapid degradation until Day 2, whereas the configuration of high levels for the relevant factors enabled the scaffold to maintain its structure until Day 23. In addition, the observed slight deviation between the degradation times of Sample 2 and Sample 3 can be attributed to a single differentiating factor, which is the UV exposure, while all other post-printing parameters remained consistent in their handling. Sample 3, which did not undergo UV treatment, displayed a slightly faster degradation rate compared to Sample 2. These findings underline the significance of considering UV exposure as a post-printing treatment factor in the DoE framework.

#### 2.2.2. Swelling Ratio

**Figure 3:**
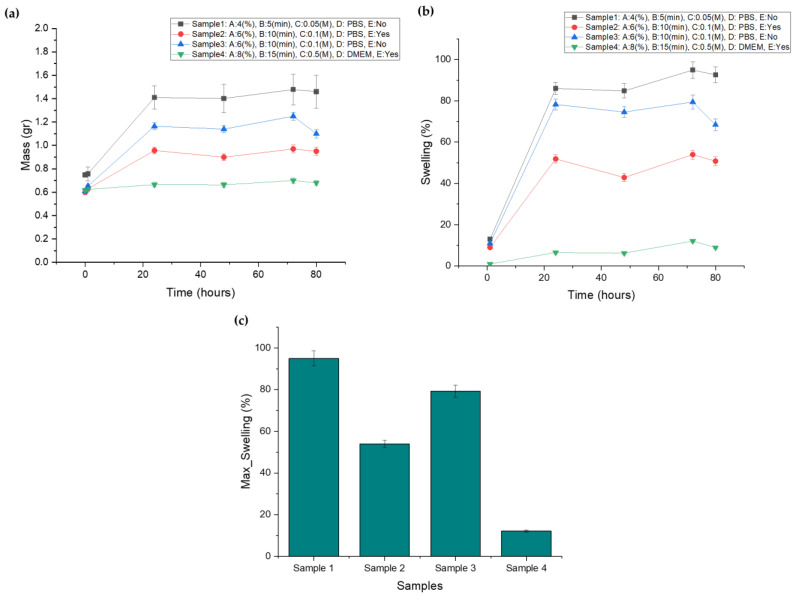
(**a**) Screening samples’ mass plot after immersion in culture media, (**b**) screening samples’ swelling curve after immersion in culture media, (**c**) maximum swelling ratio for each screening sample.

The scaffolds’ swelling behavior first reached a high absorbency and subsequently showed a decline in the swelling percentage. This constitutes a typical phenomenon for an ionically crosslinked hydrogel. When the ionic strength of the swelling medium is raised, the swelling capacity of ionic hydrogels decreases, resulting in poor anion–anion electrostatic repulsion and a decreased osmotic pressure [30]. In line with this, the plateau of the scaffolds’ maximum swelling was observed up to Day 3, regardless of the crosslinker’s concentration or the immersion time. As is evident from Figure 3a, displaying the combination of the factors, lower levels led to greater media absorption, gradually increasing the scaffolds mass. A lower alginate ratio, combined with less immersion time and a lower crosslinker concentration, lead to a limited degree of crosslinking which, in turn, resulted in a less dense hydrogel network that is able to absorb water, as recorded in the mass diagram of Sample 1 (Figure 3a), which exhibits the highest swelling ratio. On the other hand, Sample 4 represents a configuration characterized by the high levels of these factors, resulting in extensive crosslinking of the alginate blocks. This higher-level combination scaffold (Sample 4) displayed minimal deformation due to its density, which hindered media absorbance. By Day 3, Samples 1–4 displayed maximum swelling ratios of 96.543%, 79.833%, 57.120%, and 12.090%, respectively, as presented in Figure 3b and c. The value range covers the targeted swelling rate of 50.00% and is evaluated as suitable for the process optimization. The fluctuation observed in the scaffolds’ mass recording is partly attributed to temperature, which results in gelatin dissolution, and partly to the change in culture medium at Day 2. After the change in the culture medium, the process of monovalent sodium ions replacing divalent calcium ions reoccurs, leading to decreased crosslinking degree and increased swelling ability until the reestablishment of the ion equilibrium [14].

### 2.3. Design of Experiments

#### 2.3.1. Model Summary and Regression Analysis for Response 1: Degradation Time and Response 2: Swelling Ratio

The structure of the RSM’s robust design and the degradation and swelling results for each sample are presented in Table 2. The set of experiments was conducted two times and the mean value was obtained for both responses. The results acquired from the run of 34 experimental sets, suggested by the I-optimal type of RSM model, were analyzed by the Design Expert© 11.0 (Stat-Ease, Minneapolis, MN, USA) software. For the degradation time results, the day that the scaffolds were dissolved in the media, losing their original structure, was recorded while, for the swelling ratio results, the maximum swelling ratio of each run was recorded. As shown in Table 2, a maximum degradation time of 23 days was achieved with runs 24 and 29. The maximum swelling ratio of the hydrogel scaffolds was 120.3% (run 19). However, the targeted swelling ratio is 50.00%. Swelling ratios of 52.5% and 49.9% were observed in runs 9 and 27 respectively.

To evaluate whether the design is well-balanced and study the possibility of multicollinearity, the Standard Error, the Variance Inflations Factor, and the Ri^2^ values were obtained and assessed, as displayed in Table 3. As shown, the similarity and small values of standard errors indicate a balanced design and precise measurements, while VIF values of about 1 suggest no presence of multicollinearity. In addition, an Ri^2^ close to zero indicates that the terms included in the model are not interrelated, a phenomenon which could lead to suboptimal model performance [22,23].

To assess which terms and interactions among the variables should be included in the model, two-way non-parametric Kruskal–Wallis tests (ANOVA equivalent) were run. According to the *p*-values (<0.05) (Table 4), the interaction terms AB, AC, AE, BC, BD, and CD will be included in the degradation analysis model, and the interaction terms AC and BC will be, respectively, included in the swelling ratio analysis model [23].

With regard to the degradation analysis, the model is described as significant (*p*-value < 0.0001) (Table 5). A model F-value of 75.040 can also be considered as an indicator of the model’s significance. The Lack of Fit F-value of 1.819 suggests that the Lack of Fit is not statistically significant when compared to the pure error and it indicates that the model is a good fit for the data (Table 5) [23]. The R-squared value of the degradation analysis model is 0.975, indicating a very strong level of correlation. The Predicted R^2^ of 0.933 closely aligns with the Adjusted R^2^ of 0.963, with a difference of less than 0.2 between them (Table 5) [23,31,32].

As supported by the *p*-values (Table 5), among the design’s selected factors, Alginate ratio, Time, Concentration, and Media all have a significant effect on the scaffold’s degradation behavior and should be taken into account to achieve the targeted functionality. The UV Exposure term (*p*-value > 0.05) could be excluded from the analysis, leading to model reduction. However, not including this term did not increase the R^2^ value. In addition, in order to define the required steps for the optimal post-printing handling of the scaffolds, the UV exposure effect was decided to be further investigated and, thus, included in the degradation analysis model.

**Figure 4:**
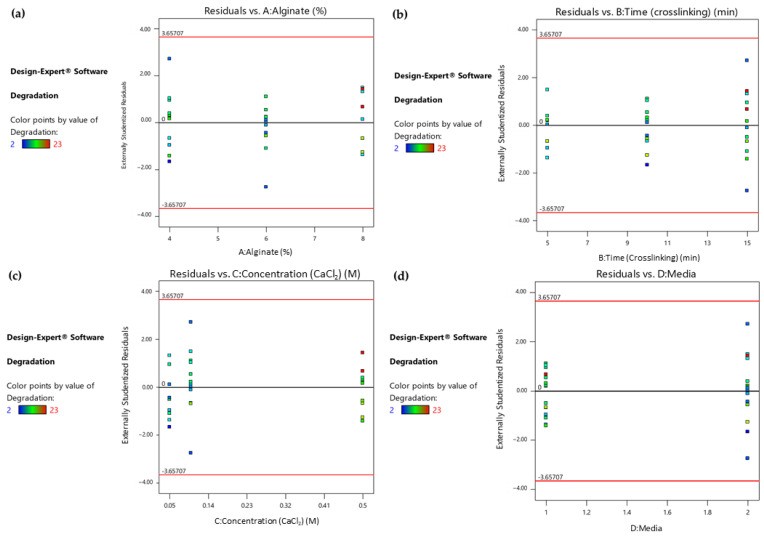
(**a**) Response 1: Residual vs. Alginate ratio plot, (**b**) Response 1: Residual vs. Time (crosslinking) plot, (**c**) Response 1: Residual vs. Concentration (CaCl_2_) plot, (**d**) Response 1: Residual vs. Culture media plot.

Furthermore, in order to identify any patterns and outliers in the residuals associated with different factor levels or combinations, the Residuals vs. Factor plots were studied. The horizontal axis of the plots in Figure 4 represents the factors that were identified as significant and their levels. As shown in the colored dot plots (Figure 4a–c), the samples that maintained their structural integrity for more days when immersed in the culture medium (marked as red points) were those that were treated with the maximum levels of the three statistically significant numerical factors. In addition, the degradation time was maximized when the samples were immersed in DMEM instead of PBS, as shown in Figure 4d. The fact that the residuals fall between the two horizontal red lines suggests that the residuals have a relatively uniform variability and are evenly distributed around zero, and that the patterns in the data are sufficiently captured.

Regarding the analysis of the scaffolds’ swelling ratios, the statistical model shows strong significance with a very low *p*-value (<0.0001) (Table 6). The Lack of Fit F-value of 2.68 implies that the Lack of Fit is not significant relative to the pure error, indicating a good fit of the model [23]. The R-squared value for the swelling ratio analysis model is 0.898, demonstrating a strong level of correlation. In addition, the Predicted R^2^ of 0.829 is in reasonable agreement with the Adjusted R^2^ of 0.870, as the difference is less than 0.2 (Table 6) [23,31,32].

Studying the *p*-values of the terms included in the model (Table 6), it is evident that the factors A, alginate ratio, and C, concentration (crosslinker), have a statistically significant impact on the swelling behavior of the samples. However, the factors B, D, and E could not be omitted from the model as the main goal of this study is to level all post-printing treatment conditions by taking into consideration the optimization of both the degradation and swelling behavior in a simultaneous manner.

**Figure 5:**
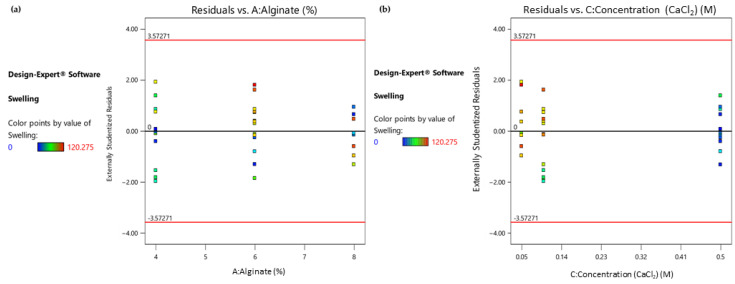
(**a**) Response 2: Residual vs. Alginate ratio plot; (**b**) Response 2: Residual vs. Concentration (CaCl_2_) plot.

As depicted in the colored dot plot (Figure 5), when studying the statistically significant factors separately, it can be deduced that the samples exhibiting a moderate swelling ratio (marked as green points) are those that had lower levels of their alginate ratio (Figure 5a) or those which were treated with a higher-level concentration of CaCl_2_. Moreover, both diagrams are indicators of a uniform variability and even distribution.

Finally, in order to understand how the values of both responses vary, a Reduced Two-Factor Interaction Model was employed to propose a possible relationship between the five most-critical post-printing variables affecting the scaffold’s functionality and the statistically significant term-interactions. Based on this, two reduced 2FI models (Table 7) were generated using the Design Expert software to forecast the degradation time (days) and swelling ratio (%). Both equations are expressed in terms of the actual factors. For this reason, the coefficients in the equation are adjusted to account for the units of each factor and should not be utilized to assess the relative influence of each factor.

**Figure 6:**
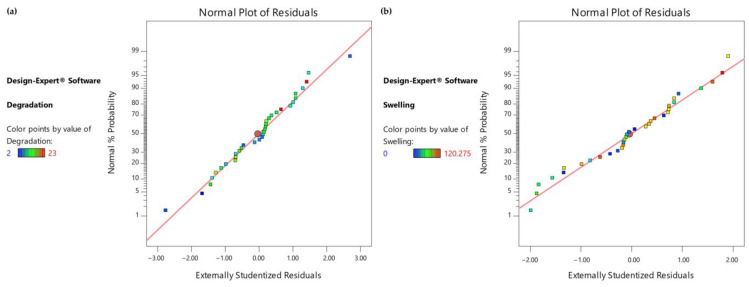
(**a**) Normal Probability Plot for degradation time observations, (**b**) Normal Probability Plot for swelling ratio observations.

As the culture medium and UV exposure treatment are two categorical predictors, the model consists of four equations, one for each of the combinations of the two categorical variables. In addition, as presented in Figure 6, as the line is not significantly skewed either left or right, it can be assumed that there is no significant deviation from the normal distribution for the observations for both responses.

In order to evaluate the equations’ accuracy, a confirmation experiment was piloted selecting the medium levels of the factors: A: alginate ratio = 6%, B: time (crosslinking) = 10 min, C: concentration (CaCl_2_) = 0.1 M, D: media = DMEM, UV exposure = Yes. The measurements were conducted in triplicate and the average values were obtained for both responses (degradation time and swelling ratio). The results and the comparison with the values predicted by the actual reduced 2FI model equations are presented in Table 8. Both experimental values fall into the Confidence Interval, indicating the equations’ high predictability.

#### 2.3.2. Parameter Optimization by Response Surface Methodology for Scaffold’s Post-Printing Treatment

When optimizing the post-printing treatment for scaffolds, specific criteria must be met to ensure their suitability for the targeted application. For instance, in the case of in vitro micro-vessel network formation, a critical evaluation of 3D cultures typically occurs at the 14-day mark. However, it is essential to note that optimal scaffold performance in facilitating vessel formation often demands structural integrity extending beyond this period, ideally reaching the 21-day threshold. Additionally, the swelling ratio of 3D systems is of pivotal importance, as excessive swelling can lead to inflammation and discomfort, while minimal deformation may result in detachment post-implantation. As a guideline, a moderate swelling ratio of 50% could strike the targeted balance. These criteria help guide the effective optimization of post-printing treatment for scaffolds, ensuring their suitability for the intended applications [6,17,21].

**Figure 7:**
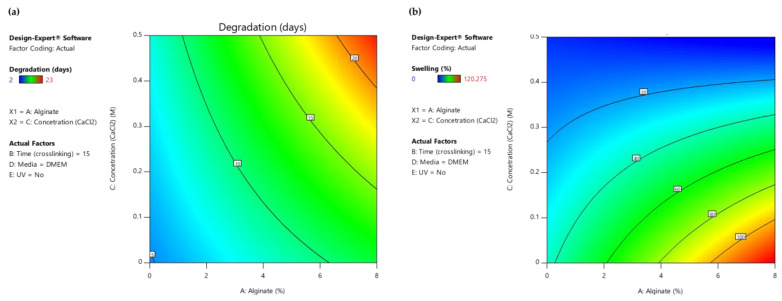
(**a**) AC Contour model graph for degradation analysis with term B fixed at 15 min, (**b**) AC Contour model graph for swelling ratio analysis with term B fixed at 15 min (Design-Expert 11 was employed).

As displayed in the contour diagrams (Figure 7), for factor B: time (crosslinking) = 15 min, the higher the alginate ratio and the crosslinker concentration, the higher the time interval that the scaffolds maintain their structure when immersed in culture medium (contoured as red region) (Figure 7a). However, for the same conditions, the swelling ratio is rather low (contoured as blue region) (Figure 7b), not reaching the targeted value of 50% (contoured as green region) (Figure 7b). For this reason, an optimization process was piloted to define the optimum compromise between the two expected functionalities.

**Figure 8:**
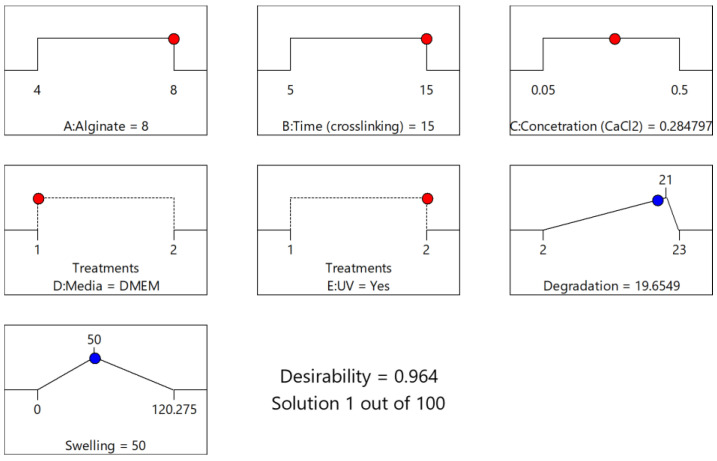
Solution’s Ramps graph (1/100; Desirability = 0.964) for optimization of both Response 1 and Response 2.

The numerical optimization [23,33,34] process was conducted with the primary objective of addressing two specific functionalities of the scaffold: achieving a low degradation rate and maintaining a moderate swelling ratio. In order to achieve these goals, the optimization criteria were precisely defined. The targeted values for the two key responses were set at 21 days for the degradation time and 50% for the swelling ratio. Through the optimization modeling process, a total of 100 solutions were generated, with their desirability decreasing progressively. Remarkably, the initial solution, which defined the optimal levels for both the numerical and categorical factors, yielded an impressive desirability score of 0.964. Implementing these recommended factor settings resulted in the model predicting a degradation time of 19.654 days and a swelling ratio of 50.00%. This achievement not only met one of the primary criteria (swelling ratio) but also exhibited nearly ideal degradation behavior. The predicted optimal values for each variable are comprehensively presented in the Ramps diagram (Figure 8) and summarized in Table 9.

**Figure 9:**
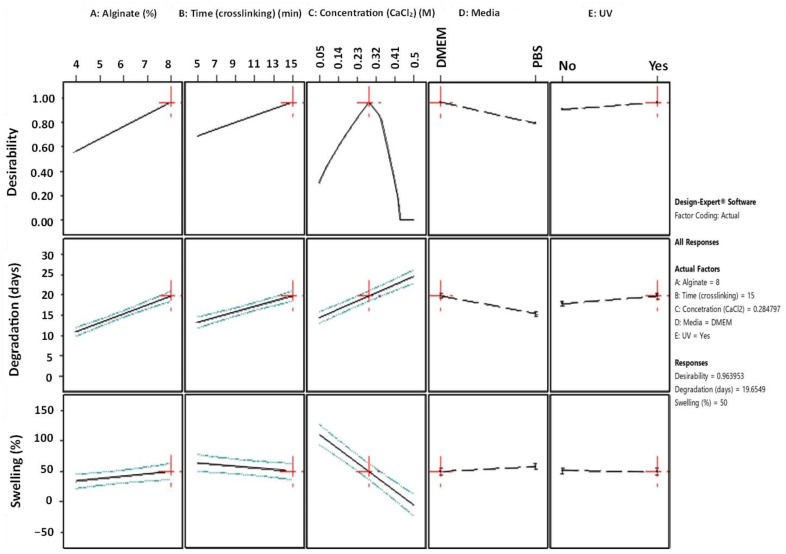
All Factors graph with annotation for 1/100 numerical optimization’s solution (Design-Expert 11 was employed).

The “All Factors” diagram (Figure 9) plots each individual response on the *y*-axis against each design factor on the *x*-axis, providing a structured representation of the relationship between variables and responses. Additionally, to encapsulate the co-optimization objective, a third response is introduced in the diagram by plotting desirability on the *y*-axis. It becomes evident that, for the majority of the responses, an apparent linearity prevails. Notably, the Desirability-Concentration (CaCl_2_) plot deviates from this pattern, revealing a non-linear relationship where the peak response is displayed at C = 0.284 M.

**Figure 10:**
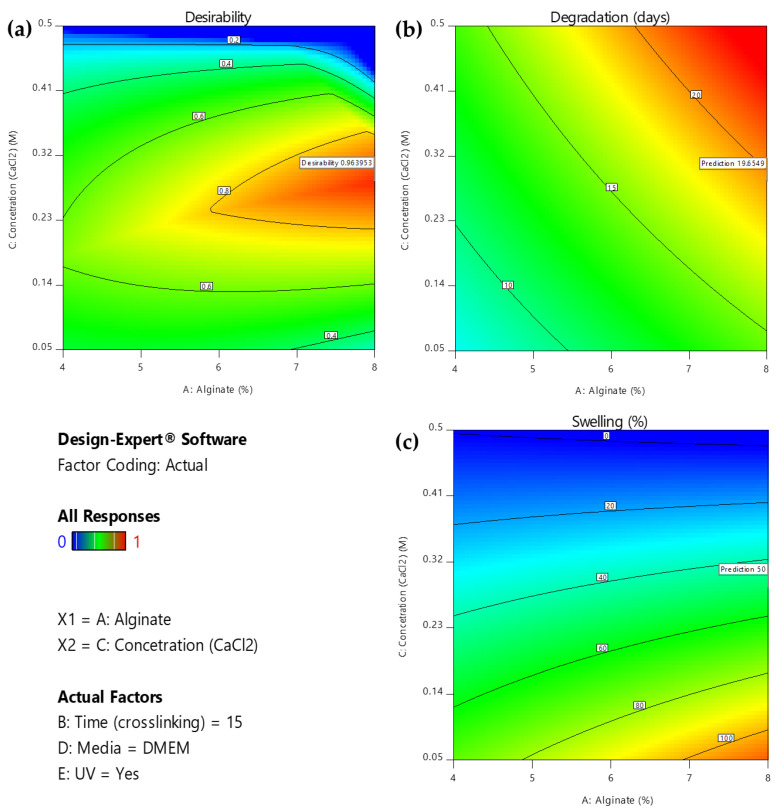
2D Contour plot for effect of the interaction of A: alginate ratio and C: concentration (CaCl_2_) factors on (**a**) desirability, (**b**) degradation, and (**c**) swelling respectively for B = 10 min. (Design-Expert 11 was employed).

Furthermore, the slope of these lines demonstrates the magnitude of a variable’s impact on the response; a steeper slope signifies a more pronounced effect. As is comprehensively presented, the optimization’s objective can be reached by using a high alginate ratio for a high level of immersion time in the medium crosslinker concentration. In regard to the conditions, UV exposure is suggested. Immersing the scaffolds in PBS might negatively affect the degradation rate of the scaffold. These results are also verified by the 2D contour plots (Figure 10), where the highest desirability is observed (contoured as red region) for high alginate ratios and medium levels of crosslinker concentration when the immersion time in DMEM is 15 min.

#### 2.3.3. Model Validation

**Figure 11:**
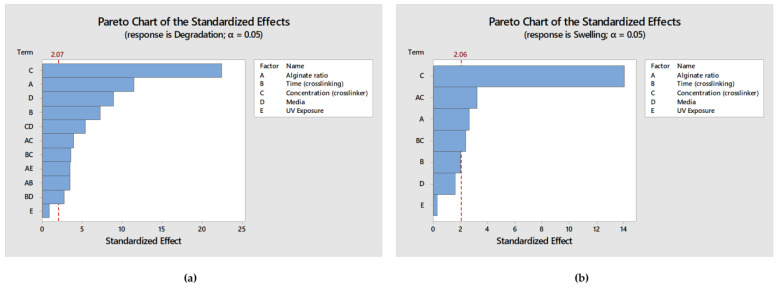
(**a**) Pareto Chart displaying the terms from the largest effect to the smallest effect on degradation response with a reference line for statistical significance; (**b**) Pareto Chart displaying the terms from the largest effect to the smallest effect on swelling ratio response with a reference line for statistical significance.

**Figure 12:**
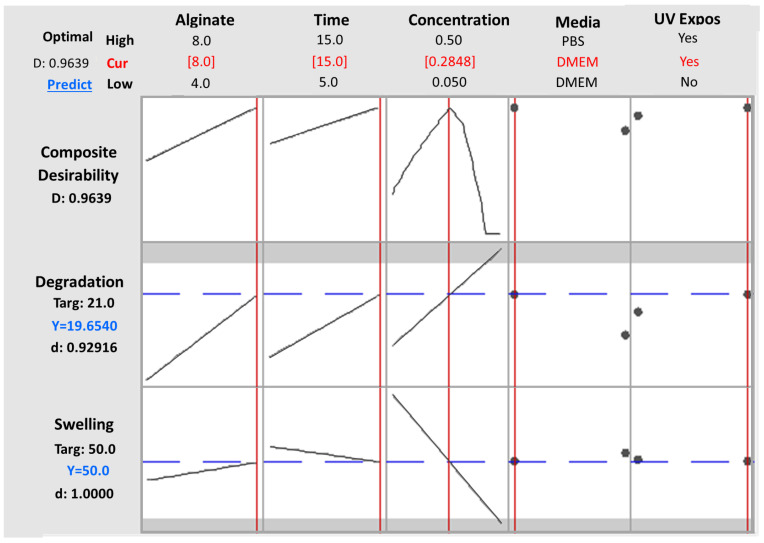
All Factors graph obtained by Minitab 2021 software, validating the predicted levels for each parameter as defined by Design Expert software for desirability = 0.9639.

In order to validate the obtained models and predicted optimal values, a parallel analysis in Minitab 2021 was conducted utilizing the levels’ configuration generated by the RSM design, along with the corresponding results for the degradation and swelling ratio. The results of this analysis in Minitab 2021totally mirrored those obtained in Design Expert (DE). Specifically, the same terms and interactions were identified as statistically significant for both the degradation and swelling ratios, as presented in Figure 11a,b respectively. Moreover, regarding the generated models’ analysis of variance as well as the models’ summary, the calculated values are identical to those obtained from DE as presented in Table 10, describing the observed data effectively. Moreover, the desirability score was again calculated as 0.963, affirming that the validation test confirmed the existence of a solution that optimizes both responses without significant compromise. Notably, as shown in Figure 12, all of the predicted optimal levels are completely identical with those obtained from DE. Thus, the results obtained from this validation approach completely align with the outcomes obtained using Design Expert, further reinforcing the accuracy of the software and models used in the study.

### 2.4. Confirmation Tests

#### 2.4.1. RSM

In order to compare the experimental results with the RSM-predicted values, confirmation experiments were then conducted by using the suggested levels of the numerical variables and by applying the optimal conditions for the post-printing treatment as indicated by the optimization process. The as-suggested treated scaffold maintained its structural integrity for 18.5 days, while reaching a swelling ratio of 54.120%. Both results are within the given limits of [18.37, 20.897] and [36.840, 63.571], respectively, according to C.I., with 95% confidence (z =1.96) (Table 11). The experimental results deviate by 5.621 (%) and 8.842 (%), respectively, from the predicted values, verifying the model’s suitability for the post-printing stage’s optimization.

Therefore, the RSM was used to define the optimum alginate ratio, time of immersion, and crosslinker concentration while determining that UV exposure facilitates the achievement of the targeted functionalities. The model also indicated that the degradation rate is higher when the scaffold is immersed in PBS, suggesting cautious and rapid procedures when treating it with PBS.

#### 2.4.2. Characterization Results for the Confirmation Tests

##### Degradation Behavior

**FT-IR Spectroscopy**

**Figure 13:**
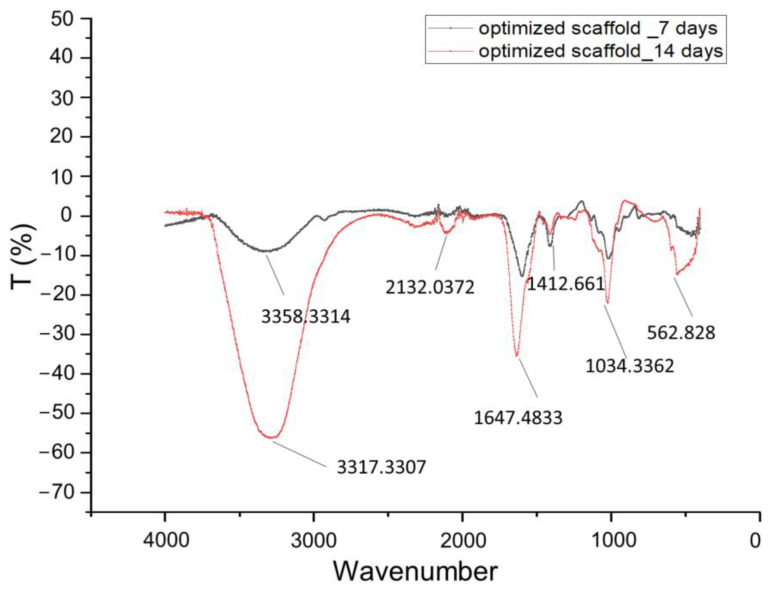
FT-IR spectra (4500–0 cm^−1^) of centrifuged culture media for optimized scaffold at Day 7 and Day 14.

Figure 13 displays the FT-IR spectra of the pellets that were collected after centrifugation of the media in which the optimized scaffold was immersed for 7 days and 14 days, respectively. The wide peak at 3358 is a characteristic peak of sodium alginate for –OH stretching [27]. The stronger peaks at 1034 (Amide III) and 1412 (O-C-C stretching) are indicative of dissolute gelatin and sodium alginate, demonstrating that, on Day 7, the scaffold exhibited lower degradation [29]. The narrow peaks at 3317 and 1647 cm^−1^, as well as at 562 and 2132 cm^−1^, are water characteristics, and this might indicate that the pellet was not totally dried. The scaffold was dissolved in the media in 18.5 days, almost reaching the ideal degradation interval of 21 days.

**Shape retention**

**Figure 14:**
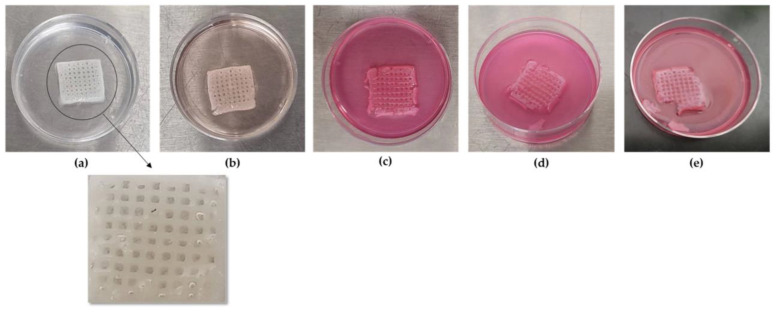
Representative photos of the scaffold were acquired at several time points. (**a**) Optimized scaffold aft post-printing treatment at Day 0, (**b**) optimized scaffold at Day 3 after immersion in DMEM, (**c**) optimized scaffold at Day 7 after immersion in DMEM, (**d**) optimized scaffold at Day 15 after immersion in DMEM, (**e**) optimized scaffold at Day 18 after immersion in DMEM.

**Figure 15:**
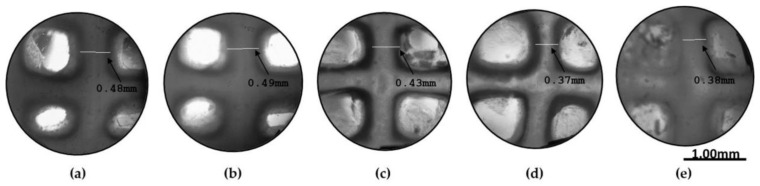
Representative images of the scaffold were acquired at several time points. (**a**) Strand retention measurements at Day 0, (**b**) strand retention measurements at Day 3, (**c**) strand retention measurements at Day 7, (**d**) strand retention measurements at Day 14, (**e**) strand retention measurements at Day 18 (Zeiss, Axiovert S100, Oberkochen, Germany) (×5 magnification).

**Figure 16:**
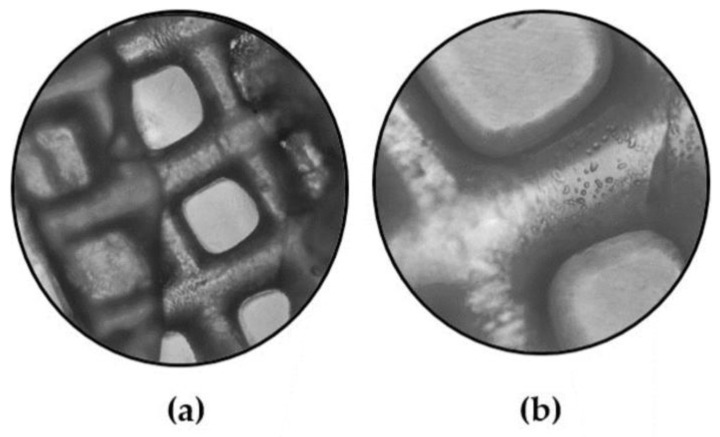
Representative images of the scaffold were also acquired also at Day 1 to ensure the scaffold’s sustainability. (**a**) Scaffold at Day 1 (×4 magnification), (**b**) scaffold at Day 1 (×10 magnification) (Zeiss, Axiovert S100, Oberkochen, Germany).

In order to more comprehensively present the degradation behavior of the optimized scaffold from Day 0 of its post-printing treatment until Day 18.5 when the scaffold lost its structural integrity, photos were taken at various time intervals as displayed in Figure 14. As is shown, the scaffold retained its infill’s structure at a sufficient level up to Day 18. For the shape retention measurement, the shape of the scaffold was also recorded at various time intervals (3, 7, 14, and 18 days) during its 18.5-day culturing (Figure 15). Representative images of the scaffold were also acquired at Day 1 to ensure the culture conditions and the sustainability of the produced 3D structure (Figure 16). By using a face contrast microscope (Zeiss, Axiovert S100, Oberkochen, Germany, 5×), images of the scaffold were obtained and analyzed via Image J software. A commonly used criterion to evaluate the shape fidelity after treatment of the scaffold is based on the measurement of the diameter of the deposited filament (strand) and its comparison with the theoretical strand diameter (0.41 mm based on the nozzle diameter).

**Figure 17:**
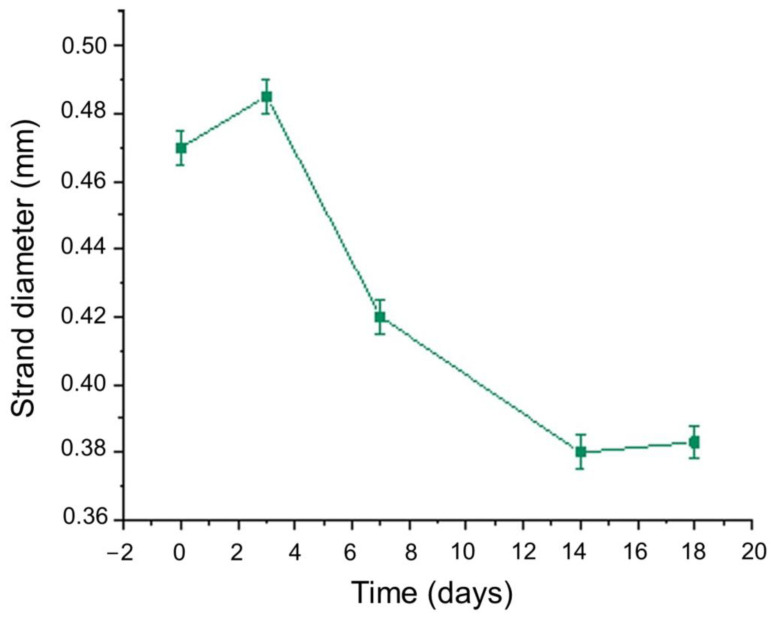
Mean value of optimized scaffold’s strand diameter versus time.

For the diameter of each strand, multiple measurements were taken and the mean value was calculated. For instance, the vertical strand’s diameters are displayed in Figure 15, indicating the scaffold’s deformation throughout the degradation study. As was expected, until Day 3, where the plateau in the swelling curve occurs, the scaffold was expanding due to water uptake, and an increase by 2.05% in the scaffold’s strand diameter was recorded. From Day 3 to Day 18.5 (Figure 17), the scaffold gradually exhibited degradation, leading to the reduction in the strand’s diameter by 39.4% before its complete dissolution in the culture media.

**Morphology**

**Figure 18:**
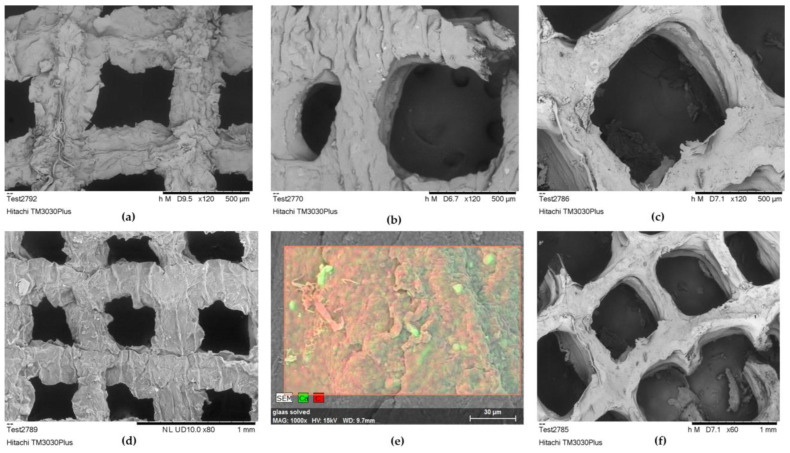
(**a**) Optimized scaffold’s infill structure integrity morphology assessment at Day 0 at an acceleration voltage of 5 kV (×120 magnification); (**b**) optimized scaffold’s infill structure integrity morphology assessment at Day 14 after immersion in DMEM, at an acceleration voltage of 5 kV (×120 magnification); (**c**) optimized scaffold’s infill structure integrity morphology assessment at Day 18 after immersion in DMEM, at an acceleration voltage of 5 kV (×120 magnification); (**d**) optimized scaffold’s infill morphology at Day 0 at an acceleration voltage of 15 kV (×80 magnification); (**e**) EDS analysis of scaffold’s infill selected region, at Day 14 after immersion in DMEM at an acceleration voltage of 15 kV (×1000 magnification); (**f**) optimized scaffold’s infill morphology at Day 18 at an acceleration voltage of 5 kV (×60 magnification).

SEM characterization was applied as a supplementary method for assessing the infill morphology of alginate–gelatin scaffolds over time, following their immersion in DMEM. In line with the shape retention results, the optimized scaffold demonstrated high stability, maintaining its infill structure integrity from Day 0 through to Day 14 and Day 18, as presented by the preserved primary shape of the pores in Figure 18a–c. However, on Day 14, a critical time point for the cell culture, a slight crack appeared in the pore’s perimeter, signifying the initial stages of gradual alteration of the scaffold’s geometry. Comparing the morphology of the Day 0 scaffold (Figure 18d) with that of Day 18 (Figure 18f), it is apparent that the pores significantly retained their geometry, while the scaffold’s macroscopic structure exhibited some deformation, likely due to gelatin dissolution. Additionally, a representative EDS analysis conducted on Day 14 ensured the detection of calcium in the infill, responsible for crosslinking the alginate blocks, despite its release into the culture media and replacement by sodium ions (Figure 18e).

##### Swelling Behavior

**Swelling Ratio**

**Figure 19:**
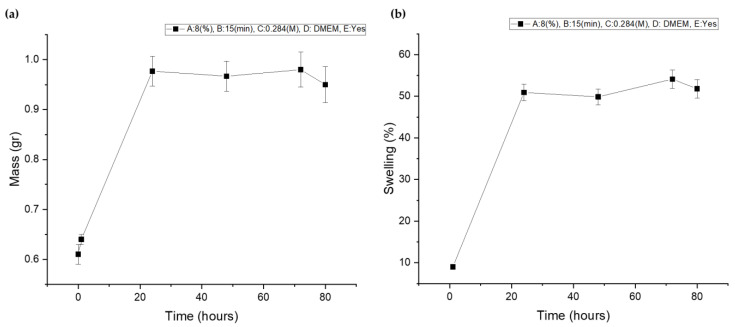
(**a**) Optimized scaffold’s mass plot after immersion in culture media; (**b**) optimized scaffold’s swelling curve after immersion in culture media.

As was expected, after the optimized level combination, the scaffold exhibited moderate deformation (Figure 19a), reaching a maximum swelling ratio of 54.120% (Figure 19b) and reaching the critical equilibrium of strong water uptake that facilitates cell viability and nutrient diffusion but not exhibiting excessive deformation that could cause inflammation or discomfort to the host after implantation. The fluctuation observed in the swelling ratio of the optimized scaffold is a result of multiple factors. Firstly, temperature variations can induce gelatin dissolution, leading to an initial degradation which was recorded at Day 2. However, the change in culture media at Day 2 triggers, again, the ion exchange, leading to a temporary decrease in the crosslinking degree and an increase in the scaffold’s swelling ability. These changes persist until the ion equilibrium is reestablished, resulting in the subsequent stabilization of the swelling ratio [14,30].

## 3. Conclusions

This study, which constitutes a follow-up to our previously published work [22], represents a systematic and data-driven exploration of post-printing treatment optimization which aimed to enhance the entire implementation of hydrogels’ DIW. The current work is driven by the use of RSM DoE to uncover the optimal combination of conditions for the desired degradation and swelling behavior of the printed structure, further advancing the field of bioprinting and tissue engineering. By employing RSM DoE, this study identified the individual and interactive effects of five post-printing treatment factors on scaffolds’ degradation time and swelling behavior, and also suggested an optimized methodology to guide the selection of post-printing treatment conditions tailored to the scaffold’s targeted functionalities.

Thus, this study managed to address a critical gap in the existing literature by concurrently optimizing the degradation and swelling behavior of DIW scaffolds. The developed model has not only identified the key factors influencing the degradation time and swelling ratio but has also yielded a solution that achieves a remarkable desirability score of 0.964, closely approaching the desired benchmarks of 21 days for degradation and a 50% swelling ratio. The optimal conditions, including an 8% alginate ratio, 15 min of crosslinking time, a crosslinker concentration of 0.284 M, DMEM as the immersion medium, and the incorporation of UV exposure, have been successfully predicted with a high degree of accuracy, with experimental values confirming the model’s reliability. Furthermore, this optimization process has shed light on crucial post-printing handling procedures for scaffolds, notably revealing that UV exposure enhances structural integrity while decreasing the degradation rate and emphasizing the need for caution when treating the scaffolds with PBS, which accelerates degradation. This innovative approach highlights the applicability of the Response Surface Methodology in simultaneous response optimization, offering a promising possibility for future research aimed at co-optimizing scaffold-targeted responses, and ultimately enhancing scaffold functionality without compromising other critical properties. This approach aims for not only improved scaffold functionality but also enhanced efficiency and resource utilization by reducing reliance on trial-and-error methods.

The present research is focused on the post-printing treatment of a specific composite hydrogel based on its common use in tissue engineering applications. Although the widespread use of alginate–gelatin hydrogel makes this study’s findings as relevant to a significant portion of ongoing research in this field, the study’s scope is limited to the materials and conditions utilized in this specific experiment and may not cover a broad range of possible materials and conditions relevant to all applications. However, the proposed approach, which entails the simultaneous optimization of two critical responses, offers a versatile and customized methodology. This adaptability enables future studies to tailor the DoE’s variables to match the specific materials and conditions in their optimization scenarios, thereby broadening the current study’s applicability across a diverse range of hydrogel-based 3D bioprinting systems. This paper introduces an innovative methodology that addresses the intricate balance between these pivotal properties and offers a novel dimension to scaffold optimization through the application of RSM.

## 4. Materials and Methods

### 4.1. Design of Experiments

The responses that this study aims to optimize are two-fold: degradation time and swelling ratio. Degradation time is a crucial factor in scaffold functionality, influencing the rate at which the scaffold provides support before naturally degrading as the vessel network is formatting [6]. Simultaneously, maintaining strong but not excessive water uptake enhances cell migration and infiltration, as well as nutrient diffusion. Achieving an optimal balance between scaffold degradation time and swelling ratio is a complex and critical task [35]. For this challenge to be addressed, this study adopts a systematic and data-driven approach utilizing Statistical Response Surface Methodology (RSM DoE). RSM DoΕ is an experimental design technique used to optimize processes, as it offers a comprehensive means to navigate the interdependent factors that influence a system’s responses.

Thus, three numerical factors were selected for investigation: alginate ratio, crosslinker concentration, and immersion time. These factors are crucial elements of the scaffolds’ final properties, affecting their structural integrity, deformation, and biocompatibility. Additionally, two categorical factors are be explored, namely UV treatment and culture media, recognizing their substantial impact on post-printing treatment outcomes.

The experimental design encompasses 4 centroids within the factor space, yielding a total of 34 runs. Each run represents a carefully orchestrated combination of these factors, allowing for a comprehensive study of the selected parameter range.

Prior to the development of the Statistical Response Surface Methodology step, a crucial screening stage was essential to qualitatively define the most influential factors affecting the response variables: *degradation time* and *swelling*. This initial phase also aimed to establish a range of values for each factor within which the optimal levels were more likely to be found. The selection of the alginate–gelatin blend composition range was guided by our previous work, which was grounded in the outcomes of the material processability optimization process [22]. In this context, the gelatin ratio was fixed at 4%, while the alginate ratio varied between 4%, 6%, and 8%. Regarding the ionic crosslinking of sodium alginate, the screening tests indicated that the two most critical factors were the concentration of the crosslinker and the immersion time of the scaffolds in the crosslinking solution. Based on the existing literature, for the initial observations of scaffold degradation behavior, the designated value range for CaCl_2_ concentration was set at [0.01–0.8] M. However, scaffolds that were treated with CaCl_2_ solutions in the range of [0.01–0.04] were difficult to handle and exhibited a very high degradation rate. On the other hand, hydrogels treated with CaCl_2_ solutions in the range of [0.6–0.8] were too dense and exhibited a very low degradation rate. For these reasons, the final range for CaCl_2_ concentration was set at [0.05–0.5]. Although the release of calcium ions from the printed structures during their culture could potentially have a cytotoxic effect, the selected high level of 0.5 M CaCl_2_ is recorded as not cytotoxic with good biocompatibility results [21]. In line with the findings of the existing literature and after screening trials, the immersion time was set to range from 5 to 15 min. Furthermore, it was of paramount importance to investigate the impact of culture media and UV treatment on the scaffold, as these two factors represent major conditions that need to be considered during the post-printing treatment of the scaffolds. With regard to culture media, DMEM is a well-established and widely utilized basal medium known for its capacity to facilitate the proliferation of diverse mammalian cell lines, offering great suitability as the selected medium for this experimental setup. The future perspectives of this study include the co-culture of cells and scaffolds in DMEM. PBS is also a commonly used buffer in everyday 3D cell-culture routine, for scaffold washes and during procedures such as trypsinization (cell splits). Thus, these two media were selected to be studied. In addition, UV treatment was used for sterilization reasons. Towards addressing these considerations, these two factors were incorporated into the experimental design as categorical variables, with different levels determined based on the respective conditions, such as “PBS or DMEM” and “UV or No UV” treatments. The levels for each numerical factor that were classified as “low”, “medium”, and “high”, and the two levels for the categorical variables, are displayed in Table 12 and Table 13, respectively.

### 4.2. Screening Tests of DoE’s Selected Levels

In the proposed methodology, an initial screening stage was incorporated consisting of experiments to evaluate the effectiveness of the levels chosen for the RSM DoE. Aiming to determine whether these selected levels warranted further optimization, a total of four samples were prepared. These samples encompassed various combinations of factors at different levels: Sample 1 involved the low-level combination of factors, Samples 2 and 3 represented medium-level combinations, and, finally, Sample 4 encompassed the high-level combination of factors. A detailed breakdown of these combinations can be found in Table 14. The samples were then evaluated in terms of degradation rate and swelling ratio.

### 4.3. Materials

Sodium alginate (alginic acid sodium salt, low viscosity, (Alfa Aesar) Thermo Fisher Scientific, TechnoBiochem, Athens, Greece), gelatin (general purpose grade, Fisher Scientific, Waltham, MA, USA), PBS (Phosphate buffered saline tablets, Fisher Bioreagents, TechLine, Athens, Greece), and calcium chloride (CaCl_2_) (Calcium Chloride Dihydrate, Riedel de Haen, Germany) were procured and used without modifications. Also, DMEM (Dulbecco’s Modified Eagle Medium) (Gibco BRL, Life Technologies, ThermoScientific, Paisley, UK) culture medium was supplemented with 10% fetal bovine serum (FBS), 1% L-glutamine, 1% sodium pyruvate, and antibiotics (Gibco BRL, Life Technologies, Thermo Scientific, Paisley, UK) [36].

### 4.4. Hydrogel Synthesis

As indicated by the Design of Experiments, alginate and gelatin were combined in the various ratios of 4/4, 6/4, and 8/4 (%) for the generation of the three hydrogel blends displayed in Table 15. For instance, the following procedures were followed to prepare 50 mL of alginate/gelatin hydrogel 4/4 (%). A total of 2 g of gelatin was dissolved in 50 mL of PBS under continuous mechanical stirring at 60 °C. Then, an amount of 2 g of alginic acid sodium salt was added to the solution followed by stirring at 5600 rpm for 1.5 h at 50 °C to ensure homogeneity. The developed composite alginate–gelatin hydrogel inks were left on a hot plate to achieve a temperature of 30 °C before the Direct Ink Writing of the scaffolds.

### 4.5. Direct Ink Writing of the Scaffolds

The 3D printing of the scaffolds was accomplished using the Bioprinter Regemat 3D BIO V1 (REGEMAT 3D S.L., Granada, Spain). The composite alginate–gelatin hydrogel inks were loaded into 5 mL syringes. Subsequently, the material was extruded from the syringe nozzle to form a continuous filament, aided by the motor-assisted piston mechanism. The hydrogel filament was deposited layer by layer, following the designed 3D blueprint [22]. The designated scaffold dimensions were specified as L:17 mm × W:17 mm × H:3.5 mm, featuring an infill pattern of orthogonal pores measuring L:1.7 mm × W:1.7 mm. In order to ensure high printability and geometric accuracy, the optimal levels of four primary (Temperature, Extrusion Speed, Nozzle Diameter, Layer Height) and four secondary (Perimeter speed, Infill Speed, Retract Speed, Travel Speed) printing parameters were implemented. These optimal parameter settings had been previously determined through a robust Taguchi design, as detailed in our earlier work on optimizing the 3D printing process. A comprehensive overview of the nine printing parameters and their respective optimal levels can be found in Table 16.

### 4.6. Crosslinking Process

A major requirement for the ink used in Direct Ink Writing of scaffolds is for it to be easily stabilized after its extrusion from the nozzle tip for the printed structure to sufficiently maintain its geometry. For this reason, the main step that governs the post-printing treatment is the crosslinking process [37]. This step is essential not only for the enhancement of the rigidity of the hydrogel-based printed scaffolds but also for the maintenance of the scaffolds’ deformation at minimal levels. In the initial screening phase, different combinations of calcium chloride (CaCl_2_) concentrations and scaffolds’ immersion time into the crosslinking solution were investigated to identify the range of the most optimal crosslinking conditions suitable for the scaffolds. Based on the results of the screening phase, following the printing process, the scaffolds were submerged in a CaCl_2_ solution with varying concentrations (0.05 M, 0.1 M, or 0.5 M) for different time intervals (5 min, 10 min, or 15 min). Subsequently, the scaffolds were removed from the CaCl_2_ solution and tapped dry. Additionally, to activate the temperature-dependent gelatin’s physical crosslinking, the scaffolds were left at room temperature (22 °C) overnight before their immersion in culture medium at 37 °C.

### 4.7. UV-Exposure

A supplementary step in the post-printing treatment is the UV exposure of the crosslinked scaffolds. As documented by Carranza et al., despite the sterilization effect, UV samples also exhibited a higher degree of dimensional stability [38]. The samples sterilized by UV radiation in particular presented the highest dimensional stability. In the direction of studying whether the UV-exposure affects the degradation behavior of the scaffolds, some of the samples were exposed to UV light before their immersion in the culture medium, while others not. For the preparation of UV scaffolds, the samples were placed in petri dishes, exposed to 254 nm UV light in a cabinet (MSC-AdvantageTM) with type-II laminar flow for 30 min, according to common sterilization protocols [39].

In consideration of environmental sustainability, the experimental procedure was designed to minimize UV light utilization, but if UV-exposure was totally avoided, then possible contaminations in the incubator and the laboratory abductor would lead to pointless repetitions of the scaffold printing that would eventually burden the environment with a higher energy footprint and waste. To accomplish this, all samples were first printed and those selected for UV exposure based on DoE runs were collectively subjected to UV light, limiting the frequency of UV lamp usage. Moreover, safety protocols were implemented to prevent any researcher exposure to UV light during the experimentation process, utilizing automatic lock system of the laboratory door during the exposure time.

### 4.8. Degradation Test

The printed scaffolds were crosslinked and treated as indicated by the matrix runs of the DoE. In order to collect data for the monitoring of the design’s selected response, degradation time, the scaffolds were then immersed in culture media after being rinsed with ethanol (90%) for sterilization. The selected media were DMEM and PBS as it is of high importance to reassure that the structure will not be dissolute at standard cell cultures conditions. DMEM is a very commonly used medium and PBS is used in everyday routine, for cell washes and during procedures such as trypsinization (cell splits) [40]. Thus, some structures were immersed in DMEM and PBS, respectively, at 37 °C, 95% humidity, in a 5% CO_2_ incubator and steady pH conditions of 7.4 [41] for varying durations (3, 7,14, and 21 days). During the degradation study, it was ensured that the scaffolds stayed submerged in the culture media. At the end of the time points, samples were retrieved and shape-retention testing of the crosslinked hydrogel scaffolds was conducted via Image J (National Institutes of Health, Bethesda, MD, USA) software. In addition, the supernatant media was centrifuged (HITACHI High Speed Refrigerated Centrifuge, CR22, Eppendorf Himac Technologies, Takeda, Japan) and the pellets’ FT-IR spectra were obtained.

### 4.9. Swelling Test

In vitro cell culture studies depend significantly on the scaffolds’ capacity to swell, as this property permits the flow of cell nutrients inside the scaffold, increasing the cell’s longevity. Moreover, a swelling test is critical as it provides an estimate of the scaffolds’ maximum volume following implantation. In this study, the swelling tests were conducted by immersing dry samples in DMEM or PBS at 37 °C. The samples were taken out at various time intervals, they were tapped dry to remove excess water and then weighed until water absorption reached saturation. The following equation was used to determine the swelling ratio of scaffold sample (Equation (2)):

(2) $$\mathrm{Swelling}\;\mathrm{ratio}=\frac{W_{1}-Wo}{Wo}$$

where *W*_1_ is the weight of the wet scaffold after immersion in the culture media (DMEM/PBS), and *W_0_* is the weight of the dry scaffold before water uptake.

### 4.10. FT-IR Spectroscopy

FTIR spectroscopy was conducted with the FTIR spectrometer (Cary 630 FTIR, Agilent Technologies, Santa Clara, CA, USA). In parallel with morphological observation through a face contrast microscopy and Scanning Electron Microscopy (SEM), FTIR spectroscopy was the characterization method that was employed in order to study the degradation rate of scaffolds, with regard to the chemical composition of the scaffold over time [42,43,44]. Aiming for maintaining aseptic conditions, this non-invasive approach to monitor scaffold dissolution was applied.

The FTIR spectra of the pellets of the centrifuged culture media for varying durations (7 and 14 days) enabled the identification of the dissolution of alginate and gelatin in the culture media. Resolution of 2 cm^−1^ was maintained in all cases. This method not only allowed for effective tracking of scaffold dissolution but also eliminated the risk of potential contamination associated with direct scaffold handling during mass or dimensional measurements.

### 4.11. Scanning Electron Microscopy

Scanning Electron Microscopy (SEM) was applied to assess the optimized scaffold’s morphology at various time points of immersion in culture media. Alginate–gelatin hydrogel scaffolds were desiccated by passing the samples through a gradation of alcohol dehydration series followed by vacuum drying and characterized using SEM at an accelerating voltage of 5 kV. Scanning electron microscopy was performed with a Hitachi TM3030 (Thermo Fisher Scientific Waltham, MA, USA) tabletop microscope, equipped with an energy dispersive X-ray spectrophotometer (EDX) system (QUANTAX 70) for the coupled analysis of chemical structure.

### 4.12. Flow Diagram

**Figure 20:**
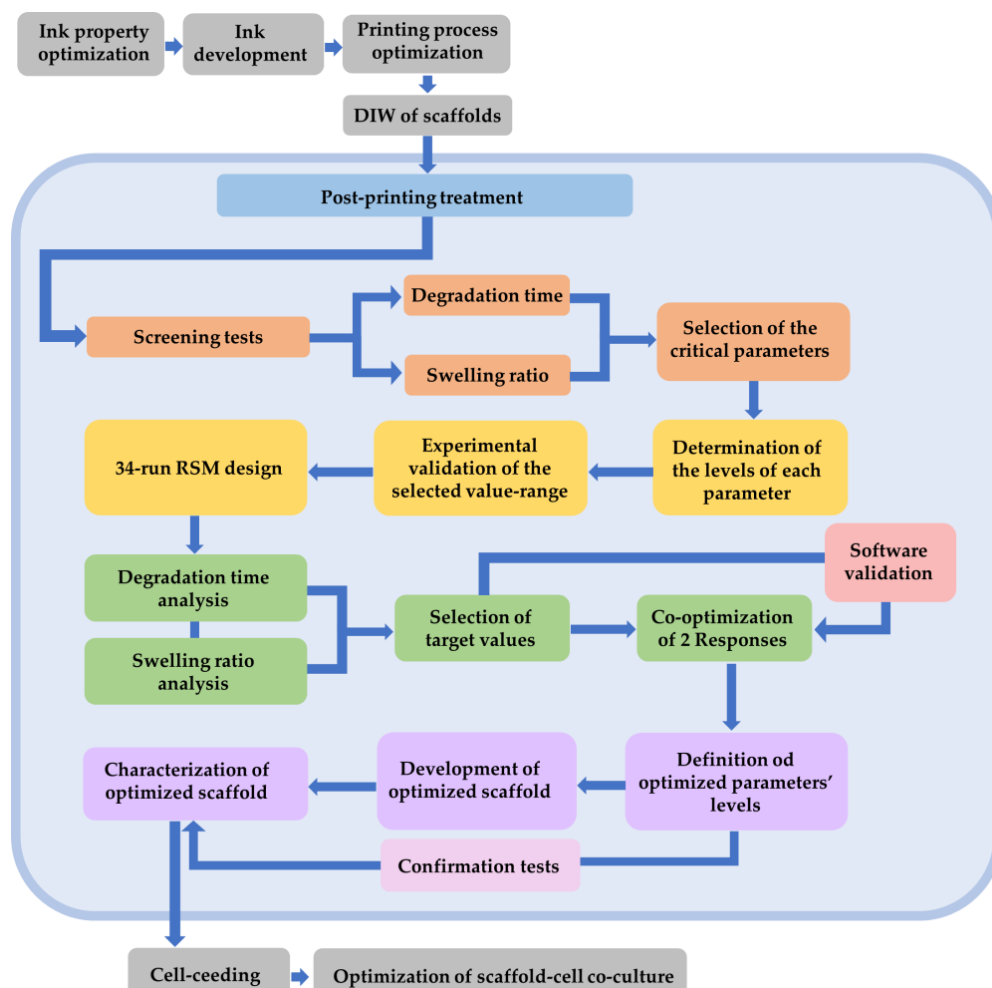
Flow diagram of the experimental procedure. The light blue area includes the main steps of this study.

In order to clarify the aforementioned methodology, the following flow diagram (Figure 20) summarizes the main steps. This study focuses on the steps that are enclosed in the light blue frame.

## Figures and Tables

**Table 1:** Scaffolds’ properties for the screening tests.

Samples	Length (mm)	Width (mm)	Height (mm)	Strand (mm)	Mass (gr)	Printability
**Sample 1**	18.121 ± 0.037	18.332±	4.061 ± 0.020	0.493 ± 0.003	0.919 ± 0.023	1.202 ± 0.035
**Sample 2**	17.793 ± 0.023	18.015±	3.465 ± 0.022	0.484 ± 0.002	0.860 ± 0.012	1.180 ± 0.019
**Sample 3**	17.924 ± 0.031	17.777±	3.791 ± 0.019	0.489 ± 0.002	0.854 ± 0.007	1.192 ± 0.017
**Sample 4**	16.767 ± 0.015	16.597±	3.374 ± 0.011	0.471 ± 0.001	0.693 ± 0.009	1.148 ± 0.011

**Table 2:** RSM Design of Experiments structure.

	Factor 1	Factor 2	Factor 3	Factor 4	Factor 5	Response 1	Response 2
**Run**	**A: Alginate**	**B: Time (CrossLinking)**	**C: Concetration (Crosslinker)**	**D: Media ^1^**	**E: UV**	**Degradation**	**Swelling**
	%	min	M	-	-	Days	%
**1**	8	10	0.5	PBS	No	16.5	16.749
**2**	6	10	0.5	DMEM	No	16	9.4675
**3**	8	15	0.05	PBS	No	7	112.544
**4**	4	15	0.5	DMEM	Yes	13	0.000
**5**	8	5	0.05	DMEM	No	7	97.262
**6**	6	10	0.1	DMEM	No	11	80.254
**7**	6	10	0.1	DMEM	No	10.5	85.993
**8**	6	15	0.05	DMEM	Yes	10	100.873
**9**	4	5	0.5	PBS	Yes	10	52.548
**10**	6	15	0.1	PBS	No	3.5	110.401
**11**	4	10	0.1	DMEM	Yes	7	39.036
**12**	4	10	0.5	DMEM	Yes	12	13.559
**13**	4	15	0.5	PBS	No	13	3.934
**14**	8	5	0.5	DMEM	Yes	16	23.195
**15**	4	5	0.05	DMEM	Yes	6	89.373
**16**	6	10	0.05	PBS	Yes	4.5	95.019
**17**	8	15	0.1	DMEM	Yes	15	82.043
**18**	4	10	0.05	PBS	No	2	88.401
**19**	6	10	0.05	PBS	Yes	4	120.275
**20**	4	5	0.5	DMEM	No	11	39.992
**21**	6	10	0.5	PBS	Yes	14.5	2.579
**22**	6	10	0.1	DMEM	No	11	91.872
**23**	6	5	0.5	PBS	No	13	25.956
**24**	8	15	0.5	DMEM	No	23	3.008
**25**	6	5	0.1	PBS	No	5	67.782
**26**	6	15	0.1	PBS	No	5.5	95.145
**27**	4	15	0.1	PBS	Yes	4	49.895
**28**	4	15	0.05	DMEM	No	9.5	71.652
**29**	8	15	0.5	PBS	Yes	23	12.945
**30**	8	10	0.1	PBS	No	6.5	105.106
**31**	6	15	0.05	DMEM	Yes	10.5	95.814
**32**	6	10	0.1	DMEM	Yes	10.5	92.279
**33**	4	10	0.1	DMEM	Yes	8.5	43.810
**34**	8	5	0.1	PBS	Yes	8	111.501

^1^ **PBS**: Phosphate buffered saline, **DMEM**: Dulbecco Modified Eagle Medium.

**Table 3:** Model terms and interactions.

Term	Standard Error *	VIF	Rᵢ^2^	Power
**A**	0.2374	1.12095	0.1079	98.0%
**B**	0.2344	1.07312	0.0681	98.1%
**C**	0.2290	1.43552	0.3034	99.6%
**D**	0.1831	1.136	0.1197	99.9%
**E**	0.1844	1.15616	0.1351	99.9%
**AB**	0.2836	1.12591	0.1118	90.9%
**AC**	0.2546	1.14246	0.1247	95.6%
**AD**	0.2421	1.14503	0.1267	97.1%
**AE**	0.2446	1.16822	0.1440	96.8%
**BC**	0.2623	1.18698	0.1575	94.5%
**BD**	0.2380	1.13322	0.1176	97.5%
**BE**	0.2398	1.14976	0.1303	97.3%
**CD**	0.1977	1.12462	0.1108	99.7%
**CE**	0.1990	1.14333	0.1254	99.7%
**DE**	0.1835	1.12884	0.1141	99.9%
**A^2^**	0.4137	1.40955	0.2906	99.5%
**B^2^**	0.4057	1.35545	0.2622	99.6%
**C^2^**	1.07	1.42195	0.2967	42.0%

* *p*-value < 0.0001 is considered as significant.

**Table 4:** Analysis of variance for interactions.

	Source	Alginate Ratio × Time	Alginate Ratio × Concentration	Alginate Ratio × Media	Alginate Ratio × UV Exposure	Time Alginate Ratio × UV Exposure Concentration	Time Alginate Ratio × UV Exposure Media	Time Alginate Ratio × UV Exposure UV Exposure	Concentration Alginate Ratio × UV Exposure Media	Concentration Alginate Ratio × UV Exposure UV Exposure	Media Alginate Ratio × UV Exposure UV Exposure
	**Terms**	**AB**	**AC**	**AD**	**AE**	**BC**	**BD**	**BE**	**CD**	**CE**	**DE**
***p*** **-Value**	**Degradation**	0.003	0.001	0.397	0.003	0.003	0.009	0.795	0.000	0.783	0.023
	**Swelling**	0.143	0.003	0.879	0.276	0.050	0.902	0.126	0.702	0.999	0.624

**Table 5:** Analysis of variance table for Response 1 and statistical summary for the linear model for the five factors affecting degradation behavior.

Source	Sum of Squares	df	Mean Square	F-Value	*p*-Value
**Model**	825.460	11	75.040	80.010	0.000
A-Alginate	124.100	1	124.100	132.300	0.000
B-Time (crosslinking)	49.550	1	49.550	52.830	0.000
C-Concentration (CaCl_2_)	474.300	1	474.300	505.670	0.000
D-Media	74.980	1	74.980	79.940	0.000
E-UV	0.659	1	0.659	0.703	0.410
AB	11.640	1	11.640	12.410	0.001
AC	14.170	1	14.170	15.110	0.000
AE	11.680	1	11.680	12.450	0.001
BC	12.140	1	12.140	12.950	0.001
BD	7.330	1	7.330	7.820	0.010
CD	27.220	1	27.220	29.020	0.000
**Residual**	20.640	22	0.938		
Lack of Fit	17.090	16	1.070	1.810	0.238
Pure Error	3.540	6	0.590		
**Cor Total**	846.100	33			
**Std. Dev.**	0.968		**R^2^**	0.975	
**Mean**	10.220		**Adjusted R^2^**	0.963	
**C.V.%**	9.480		**Predicted R^2^**	0.933	
			**Adeq. Precision**	35.370	

**Table 6:** Analysis of variance table for Response 2 and statistical summary for the linear model for the five factors affecting swelling ratio.

Source	Sum of Squares	df	Mean Square	F-Value	*p*-Value
**Model**	46,294.00	7	6613.530	32.800	0.000
A-Alginate	1384.170	1	1384.170	6.870	0.014
B-Time (crosslinking)	806.930	1	806.930	4.000	0.056
C-Concentration (CaCl_2_)	39,930.220	1	39,930.220	198.050	0.000
D-Media	528.260	1	528.260	2.620	0.117
E-UV	13.720	1	13.720	0.068	0.796
AC	2068.080	1	2068.080	10.260	0.003
BC	1150.540	1	1150.540	5.710	0.024
**Residual**	5242.070	26	201.620		
Lack of Fit	4715.090	20	235.750	2.680	0.112
Pure Error	526.980	6	87.830		
**Cor Total**	51,536.770	33			
**Std. Dev.**	14.200		**R^2^**	0.898	
**Mean**	62.650		**Adjusted R^2^**	0.870	
**C.V.%**	22.660		**Predicted R^2^**	0.829	
			**Adeq. Precision**	18.048	

**Table 7:** Degradation time equations and swelling ratio equations of the reduced 2FI model for each combination of media and UV exposure.

Degradation		Swelling	
** Media**	** DMEM**	** Media**	**DMEM**
** UV**	** No**	** UV**	**No**
+9.856		+26.131	
−0.608	Alginate	+10.985	Alginate
−0.334	Time (crosslinking)	+0.724	Time (crosslinking)
−5.645	Concentration (CaCl_2_)	+47.471	Concentration (CaCl_2_)
+0.095	Alginic × Time (crosslinking)	−24.374	Alginic × Concentration (CaCl_2_)
+2.050	Alginic × Concentration (CaCl_2_)	−7.390	Time (crosslinking) × Concentration (CaCl_2_)
+0.781	Time (crosslinking) × Concentration (CaCl_2_)		
**Media**	**DMEM**	**Media**	**DMEM**
**UV**	**Yes**	**UV**	**Yes**
+5.331		+24.838	
+0.194	Alginate	+10.985	Alginate
−0.334	Time (crosslinking)	+0.724	Time (crosslinking)
−5.645	Concentration (CaCl_2_)	+47.471	Concentration (CaCl_2_)
+0.095	Alginic × Time (crosslinking)	−24.374	Alginic × Concentration (CaCl_2_)
+2.050	Alginic × Concentration (CaCl_2_)	−7.390	Time (crosslinking) × Concentration (CaCl_2_)
+0.781	Time (crosslinking) × Concentration (CaCl_2_)		
**Media**	**PBS**	**Media**	**PBS**
**UV**	**No**	**UV**	**No**
+6.744		+34.178	
−0.608	Alginate	+10.985	Alginate
−0.585	Time (crosslinking)	+0.724	Time (crosslinking)
+3.440	Concentration (CaCl_2_)	+47.471	Concentration (CaCl_2_)
+0.095	Alginic × Time (crosslinking)	−24.374	Alginic × Concentration (CaCl_2_)
+2.050	Alginic × Concentration (CaCl_2_)	−7.390	Time (crosslinking) × Concentration (CaCl_2_)
+0.781	Time (crosslinking) × Concentration (CaCl_2_)		
**Media**	**PBS**	**Media**	**PBS**
**UV**	**Yes**	**UV**	**Yes**
+2.219		+32.885	
+0.194	Alginate	+10.985	Alginate
−0.585	Time (crosslinking)	+0.724	Time (crosslinking)
+3.440	Concentration (CaCl_2_)	+47.471	Concentration (CaCl_2_)
+0.095	Alginic × Time (crosslinking)	−24.374	Alginic × Concentration (CaCl_2_)
+2.050	Alginic × Concentration (CaCl_2_)	−7.390	Time (crosslinking) × Concentration (CaCl_2_)
+0.781	Time (crosslinking) × Concentration (CaCl_2_)		

**Table 8:** Reduced 2FI model equations’ predicted responses for numerical factors’ medium levels and observed results from confirmation tests.

Response	Predicted Mean	Predicted Median	Observed	Std. Dev	SE Mean	95% CI Low for Mean	95% CI High for Mean	95% TI Low for 99% Pop	95% TI High for 99% Pop
Degradation	12.990	12.990	14.000	0.968	0.543	11.866	14.115	8.725	17.252
Swelling	97.813	97.813	93.872	14.199	6.587	84.272	111.355	39.055	156.52

**Table 9:** RSM’s numerical optimization variable levels (Solution 1/100; Desirability = 0.964).

Factors	A: Alginate Ratio (%)	B: Time (Crosslinking)(min)	C: Concentration (CaCl_2_) (M)	D: Media (-)	E: UV Exposure (-)
**Optimal Levels**	8.000	15.000	0.284	DMEM	Yes

**Table 10:** Analysis of variance and model summary comparison as obtained by Design Expert and Minitab software, respectively.

		Source	Sum of Squares	df	Mean Square	F-Value	*p*-Value
**Degradation** **Analysis**	**Design Expert**	**Model**	825.460	11	75.040	80.010	0.000
	**Minitab**		825.460	11	75.040	80.010	0.000
**Swelling ratio Analysis**	**Design Expert**	**Model**	46,294.700	7	6613.530	32.800	0.000
	**Minitab**		46,294.700	7	6613.530	32.800	0.000
**Degradation** **Analysis**	**Design Expert**	**Std. Dev.**	**R^2^**	**Adjusted R^2^**		**Predicted R^2^**	
	**Minitab**	0.968	0.975	0.963		0.933	
**Swelling ratio Analysis**	**Design Expert**	0.968	0.975	0.963		0.933	
	**Minitab**	14.200	0.898	0.870		0.829	

**Table 11:** RSM’s DoE point prediction for optimum variable levels and observed results from confirmation tests.

Optimal Response	Predicted Mean	Predicted Median	Observed	Std Dev	SE Mean	95% CI Low for Mean	95% CI High for Mean	95% TI Low for 99% Pop	95% TI High for 99% Pop
Degradation	19.654	19.654	18.500	0.968	0.607	18.376	20.897	15.262	24.011
Swelling	50.00	50.000	54.120	14.189	6.502	36.840	63.571	−8.406	108.818

**Table 12:** Defined levels for all three numerical factors of the post-printing treatment of alginate–gelatin scaffolds.

Post-Printing Treatment Factor	A: Alginate Ratio (%)	B: Time (CrossLinking) (min)	C: Concentration (CrossLinker) (M)
Low Level	4	5	0.05
Medium Level	6	10	0.1
High Level	8	15	0.5

**Table 13:** Defined levels for both vategorical variables of the post-printing treatment of alginate-gelatin scaffolds.

Post-Printing Treatment Factors	D: Culture Media	E: UV Exposure
Level 1	DMEM	NO
Level 2	PBS	YES

**Table 14:** Factors’ level combinations for screening tests samples.

Post-Printing Treatment Factors	A: Alginate Ratio (%)	B: Time (Crosslinking) (min)	C: Concentration (CaCl_2_) (M)	D: Media (-)	E: UV Exposure (-)
**Sample 1**	4	5	0.05	PBS	No
**Sample 2**	6	10	0.1	PBS	Yes
**Sample 3**	6	10	0.1	PBS	No
**Sample 4**	8	15	0.5	DMEM	Yes

**Table 15:** Different ratios of developed alginate–gelatin hydrogel blends.

Hydrogel	Alginate (%)	Gelatin (%)
1	4	4
2	6	4
3	8	4

**Table 16:** Optimal levels for 8 selected printing settings for high printing accuracy.

Printing Parameters	Temperature (˚C)	Extrusion Speed (mm/s)	Nozzle Diameter (mm)	Layer Height (mm)	Perimeter Speed (mm/s)	Infill Speed (mm/s)	Retract Speed (mm/s)	Travel Speed (mm/s)
Printability Window	24	2	0.41	0.25	3	2	30	50

## Data Availability

The data presented in this study are openly available in article.

## References

[1] Saadi M.A.S.R., Maguire A., Pottackal N.T., Thakur M.S.H., Ikram M.M., Hart A.J., Ajayan P.M., Rahman M.M. (2022). Direct Ink Writing: A 3D Printing Technology for Diverse Materials. Adv. Mater..

[2] Gu Z., Fu J., Lin H., He Y. (2019). 3D Bioprinting: A Novel Avenue for Manufacturing Tissues and Organs. Engineering.

[3] Jiang Z., Diggle B., Li Tan M., Viktorova J., Bennett C.W., Connal L.A. (2020). Extrusion 3D Printing of Polymeric Materials with Advanced Properties. Adv. Sci..

[4] Zielińska A., Karczewski J., Eder P., Kolanowski T., Szalata M., Wielgus K., Szalata M., Kim D., Shin S.R., Słomski R. (2023). Scaffolds for drug delivery and tissue engineering: The role of genetics. J. Control Release.

[5] Saini G., Segaran N., Mayer J.L., Saini A., Albadawi H., Oklu R. (2021). Applications of 3D Bioprinting in Tissue Engineering and Regenerative Medicine. J. Clin. Med..

[6] Yavvari P., Laporte A., Elomaa L., Schraufstetter F., Pacharzina I., Daberkow A.D., Hoppensack A., Weinhart M. (2022). 3D-Cultured Vascular-Like Networks Enable Validation of Vascular Disruption Properties of Drugs In Vitro. Front. Bioeng. Biotechnol..

[7] Di Giuseppe M., Law N., Webb B., Macrae R.A., Liew L.J., Sercombe T.B., Dilley R.J., Doyle B.J. (2018). Mechanical behaviour of alginate–gelatin hydrogels for 3D bioprinting. J. Mech. Behav. Biomed. Mater..

[8] Lagopati N., Pavlatou E.A. (2020). Advanced Applications of Biomaterials Based on Alginic Acid. Am. J. Biomed. Sci..

[9] Anitua E., Zalduendo M., Troya M., Erezuma I., Lukin I., Hernáez-Moya R., Orive G. (2022). Composite alginate–gelatin hydrogels incorporating PRGF enhance human dental pulp cell adhesion, chemotaxis and proliferation. Int. J. Pharm..

[10] Lagopati N., Pippa N., Gatou M.-A., Papadopoulou-Fermeli N., Gorgoulis V.G., Gazouli M., Pavlatou E.A. (2023). Marine-Originated Materials and Their Potential Use in Biomedicine. Appl. Sci..

[11] Tomić S.L., Babić Radić M.M., Vuković J.S., Filipović V.V., Nikodinovic-Runic J., Vukomanović M. (2023). Alginate-Based Hydrogels and Scaffolds for Biomedical Applications. Mar. Drugs.

[12] Ketabat F., Maris T., Duan X., Yazdanpanah Z., Kelly M.E., Badea I., Chen X. (2023). Optimization of 3D Printing and in Vitro Characterization of Alginate/Gelatin Lattice and Angular Scaffolds for Potential Cardiac Tissue Engineering. Front. Bioeng. Biotechnol..

[13] Wierzbicka A., Bartniak M., Rosińska K., Bociąga D. (2022). Optimization of the preparation process stages of the bioink compositions based on sodium alginate and gelatin to improve the viability of biological material contained in hydrogel 3D printouts. Eng. Biomater..

[14] Chawla D., Kaur T., Joshi A., Singh N. (2020). 3D Bioprinted Alginate–gelatin Based Scaffolds for Soft Tissue Engineering. Int. J. Biol. Macromol..

[15] Mondal A., Gebeyehu A., Miranda M., Bahadur D., Patel N., Ramakrishnan S., Rishi A.K., Singh M. (2019). Characterization and printability of Sodium alginate -Gelatin hydrogel for bioprinting NSCLC co-culture. Sci. Rep..

[16] Sarker M., Izadifar M., Schreyer D., Chen X. (2018). Influence of ionic crosslinkers (Ca^2+^/Ba^2+^/Zn^2+^) on the mechanical and biological properties of 3D Bioplotted Hydrogel Scaffolds. J. Biomater. Sci. Polym. Ed..

[17] Shi L., Xiong L., Hu Y., Li W., Chen Z., Liu K., Zhang X. (2018). Three-Dimensional Printing Alginate/Gelatin Scaffolds as Dermal Substitutes for Skin Tissue Engineering. Polym. Eng. Sci..

[18] Amr M., Dykes I., Counts M., Kernan J., Mallah A., Mendenhall J., Van Wie B., Abu-Lail N., Gozen B.A. (2021). 3D Printed, Mechanically Tunable, Composite Sodium Alginate, Gelatin and Gum Arabic (SA-GEL-GA) Scaffolds. Bioprinting.

[19] Naghieh S., Karamooz-Ravari M.R., Sarker M.D., Karki E., Chen X. (2018). Influence of crosslinking on the mechanical behavior of 3D printed alginate scaffolds: Experimental and numerical approaches. J. Mech. Behav. Biomed. Mater..

[20] Bahrami N., Farzin A., Bayat F., Goodarzi A., Salehi M., Goodarzi A., Salehi M., Karimi R., Mohamadnia A., Parhiz A. (2019). Optimization of 3D Alginate Scaffold Properties with Interconnected Porosity Using Freeze-drying Method for Cartilage Tissue Engineering Application. Arch. Neurosci..

[21] Sonaye S.Y., Ertugral E.G., Kothapalli C.R., Sikder P. (2022). Extrusion 3D (Bio)Printing of Alginate–gelatin-Based Composite Scaffolds for Skeletal Muscle Tissue Engineering. Materials.

[22] Kaliampakou C., Lagopati N., Charitidis C.A. (2023). Direct Ink Writing of Alginate–Gelatin Hydrogel: An Optimization of Ink Property Design and Printing Process Efficacy. Appl. Sci..

[23] Gupta P., Nayak K.K. (2016). Optimization of Keratin/Alginate Scaffold Using RSM and Its Characterization for Tissue Engineering. Int. J. Biol. Macromol..

[24] Pepelnjak T., Stojšić J., Sevšek L., Movrin D., Milutinović M. (2023). Influence of Process Parameters on the Characteristics of Additively Manufactured Parts Made from Advanced Biopolymers. Polymers.

[25] Gao T., Gillispie G.J., Copus J.S., Pr A.K., Seol Y.J., Atala A., Yoo J.J., Lee S.J. (2018). Optimization of gelatin-alginate composite bioink printability using rheological parameters: A systematic approach. Biofabrication.

[26] Shan Y., Li C., Wu Y., Li Q., Liao J. (2019). Hybrid Cellulose Nanocrystal/Alginate/Gelatin Scaffold with Improved Mechanical Properties and Guided Wound Healing. RSC Adv..

[27] Aljohani W., Ullah M.W., Li W., Shi L., Zhang X., Yang G. (2018). Three-Dimensional Printing of Alginate–gelatin-Agar Scaffolds Using Free-Form Motor Assisted Microsyringe Extrusion System. J. Polym. Res..

[28] Li Y., Jia H., Cheng Q., Pan F., Jiang Z. (2011). Sodium alginate–gelatin polyelectrolyte complex membranes with both high water vapor permeance and high permselectivity. J. Membr. Sci..

[29] Helmiyati, Aprilliza M. (2017). Characterization and properties of sodium alginate from brown algae used as an ecofriendly superabsorbent. IOP Conf. Ser. Mater. Sci. Eng..

[30] Gupta N.V., Shivakumar H.G. (2012). Investigation of Swelling Behavior and Mechanical Properties of a pH-Sensitive Superporous Hydrogel Composite. Iran. J. Pharm. Res..

[31] Talaei A., O’Connell C.D., Sayyar S., Maher M., Yue Z., Choong P.F., Wallace G.G. (2023). Optimizing the composition of gelatin methacryloyl and hyaluronic acid methacryloyl hydrogels to maximize mechanical and transport properties using response surface methodology. J. Biomed. Mater. Res. B Appl. Biomater..

[32] Coşkun S., Akbulut S.O., Sarıkaya B., Çakmak S., Gümüşderelioğlu M. (2022). Formulation of Chitosan and Chitosan-nanoHAp Bioinks and Investigation of Printability with Optimized Bioprinting Parameters. Int. J. Biol. Macromol..

[33] El Magri A., El Mabrouk K., Vaudreuil S., Ebn Touhami M. (2021). Experimental Investigation and Optimization of Printing Parameters of 3D Printed Polyphenylene Sulfide through Response Surface Methodology. J. Appl. Polym. Sci..

[34] Vates U.K., Kanu N.J., Gupta E., Singh G.K., Daniel N.A., Sharma B.P. (2021). Optimization of FDM 3D Printing Process Parameters on ABS Based Bone Hammer Using RSM Technique. IOP Conf. Ser. Mater. Sci. Eng..

[35] Dhote V., Vernerey F.J. (2014). Mathematical model of the role of degradation on matrix development in hydrogel scaffold. Biomech. Model. Mechanobiol..

[36] Lagopati N., Kotsinas A., Veroutis D., Evangelou K., Papaspyropoulos A., Arfanis M., Falaras P., Kitsiou P.V., Pateras I., Bergonzini A. (2021). Biological Effect of Silver-modified Nanostructured Titanium Dioxide in Cancer. Cancer Genom. Proteom..

[37] Fu Q., Saiz E., Tomsia A.P. (2011). Direct ink writing of highly porous and strong glass scaffolds for load-bearing bone defects repair and regeneration. Acta Biomater..

[38] Carranza T., Zalba-Balda M., Baraibar M.J.B., de la Caba K., Guerrero P. (2022). Effect of sterilization processes on alginate/gelatin inks for three-dimensional printing. Int. J. Bioprint..

[39] Yen S., Sokolenko S., Manocha B., Blondeel E.J., Aucoin M.G., Patras A., Daynouri-Pancino F., Sasges M. (2014). Treating cell culture media with UV irradiation against adventitious agents: Minimal impact on CHO performance. Biotechnol. Prog..

[40] Katifelis H., Nikou M.-P., Mukha I., Vityuk N., Lagopati N., Piperi C., Farooqi A.A., Pippa N., Efstathopoulos E.P., Gazouli M. (2022). Ag/Au Bimetallic Nanoparticles Trigger Different Cell Death Pathways and Affect Damage Associated Molecular Pattern Release in Human Cell Lines. Cancers.

[41] Papadopoulou-Fermeli N., Lagopati N., Pippa N., Sakellis E., Boukos N., Gorgoulis V.G., Gazouli M., Pavlatou E.A. (2023). Composite Nanoarchitectonics of Photoactivated Titania-Based Materials with Anticancer Properties. Pharmaceutics.

[42] Celina M., Ottesen D.K., Gillen K.T., Clough R.L. (1997). FTIR emission spectroscopy applied to polymer degradation. Polym. Degrad. Stab..

[43] Leroy A., Ribeiro S., Grossiord C., Alves A., Vestberg R.H., Salles V., Brunon C., Gritsch K., Grosgogeat B., Bayon Y. (2017). FTIR microscopy contribution for comprehension of degradation mechanisms in PLA-based implantable medical devices. J. Mater. Sci. Mater. Med..

[44] Langueh C., Changotade S., Ramtani S., Lutomski D., Rohman G. (2021). Combination of in Vitro Thermally-Accelerated Ageing and Fourier-Transform Infrared Spectroscopy to Predict Scaffold Lifetime. Polym. Degrad. Stab..

